# Tuning the rheostat of immune gene translation

**DOI:** 10.1007/s44154-023-00087-0

**Published:** 2023-04-06

**Authors:** Shuai Huang

**Affiliations:** Department of Molecular Genetics, The Ohio State University, Columbus, OH 43210 USA

**Keywords:** Plant immunity, Liquid-liquid phase separation, Biomolecular condensates, Gene translation, Cell death, *Arabidopsis*

## Abstract

Biomolecular condensates assembled through phase transitions regulate diverse aspects of plant growth, development, and stress responses. How biomolecular condensates control plant immunity is poorly understood. In *Nature Plants*, a new study (Zhou et al., Nat Plants 9:289–301, 2023) reveals how plants assemble translational condensates to balance tissue health and disease resistance.

In electrical devices, a rheostat controls the intensity of current and ensures proper circuit functioning by adjusting its resistance. In biological systems, similar mechanisms exist to control the level of gene products and the strength of signaling output, which, if overproduced, can compromise an organism's general fitness. One of the key mechanisms that cells employ to regulate gene expression is translational reprogramming (Spriggs et al. 2010). This process allows cells to rapidly adjust the translation of specific mRNAs in response to changing environmental conditions, developmental cues, or cellular stresses. In a recent article in *Nature Plants*, Zhou et al. (Zhou et al. 2023) have begun to unravel the molecular mechanisms of this fundamental layer of immune control in the model organism *Arabidopsis thaliana* (Fig. 1).

**Fig. 1:**
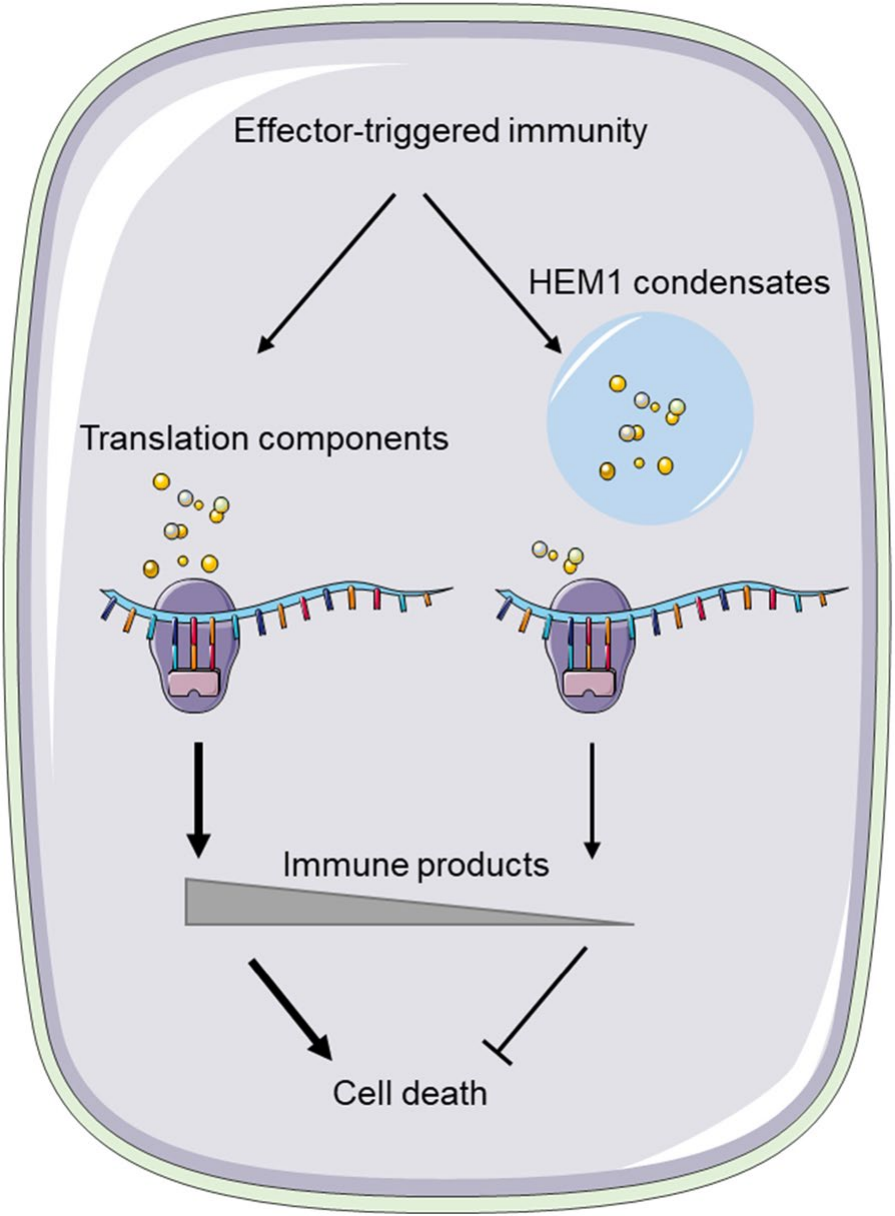
ETI-induced HEM1 condensates sequester translational components to inhibit immune gene translation and to promote cell survival. During effector triggered immunity (ETI), plants massively reprogram the translatome to produce immune gene products to restrict pathogen growth through programmed cell death. Zhou et al. found that ETI activation induces HEM1, a low complexity domain-containing protein, to form cytoplasmic biomolecular condensates through biological phase separation that sequester translation factors to promote cell survival and tissue health. This enables plants to fine-tune growth-defense trade-offs upon infectious diseases

To identify the *bona fide* regulators of global translational reprograming in plant defense, Zhou and colleagues enlisted a genetic screen employing a translationally controlled luciferase reporter system (Xu et al. 2017) to screen for mutants with enhanced luciferase activity, which served as a proxy for immune activation. The authors delineated functions of the HEM1 gene uncovered from the screen in immune responses and translational control. Pathogen infection assays revealed that HEM1 predominantly contributed to effector-triggered immunity (ETI) but not pattern-triggered immunity (PTI) (Jones and Dangl 2006). Remarkably, ETI-induced hypersensitive response (HR) cell death was greatly accelerated in *hem1* loss-of-function mutant. Hence, HEM1 served as a negative regulator of ETI pathway.

How does HEM1 repress ETI? Earlier work proposed a role for HEM1 in actin nucleation in both plants and animals (Hummel et al. 2000; Brembu et al. 2004). Zhou et al. found that HEM1 has a novel role in regulating translation efficiency. Ribosome footprinting (Ribo-seq) uncovered a global defect in translational reprogramming during immune activation. Closer inspection of Ribo-seq profiles revealed that the translation of pro-death genes was inhibited during ETI. These pro-death genes, when overproduced, cause accelerated cell death as seen for certain autoimmune mutants (van Wersch et al. 2016) and thus are under tight negative regulation in the absence of pathogen infections. To probe the molecular basis for suppression of ETI by HEM1, Zhou and colleagues enlisted protein interaction network mapping and in silico analyses. HEM1 was found to interact extensively with components of the translation machinery. Surprisingly, a plant-specific low-complexity domain (LCD) was detected at the C-terminus of HEM1. Truncation and mutagenesis assays further supported the functional role of the LCD in mediating the interaction of HEM1 with the translation machinery and repressing cell death during ETI.

Recent studies suggest that LCD-mediated liquid–liquid phase separation (LLPS) contributes to the formation of biomolecular condensates (Molliex et al. 2015), which are membrane-less assemblies that can concentrate proteins and nucleic acids to regulate diverse cellular processes (Banani et al. 2017). Zhou and colleagues tested this possibility. Biochemically, recombinant HEM1 protein underwent robust phase separation in vitro due to multivalent interactions mediated by its LCD. *In planta*, HEM1 cytoplasmic condensates were largely induced by ETI activation as revealed by live cell imaging. The authors further isolated HEM1 condensates from live plants and showed that they sequestered numerous translation factors, likely limiting their availability for the translation of pro-death genes. Phase separation-deficient HEM1 mutants failed to control ETI-induced cell death and resulted in altered translation. Clearly, HEM1 condensates are functionally important in promoting cell survival during infections. Future research should address the molecular mechanism of selectivity in client components and potential RNA involvement of HEM1 condensates during plant defense.

So, what can we learn from this study? First, it reinforces the emerging roles of biomolecular condensates in plant organismal defense (Zavaliev et al. 2020; Huang et al. 2021; Kim et al. 2022). The discovery of a condensation domain within HEM1 opens up new avenues for future research into the mechanisms underlying translational condensate formation and function in plants. Second, it provides new insights into the regulation of immune gene translation in eukaryotes. As biomolecular condensates play a critical role in a wide range of biological processes in other organisms, including neurodegenerative diseases in humans, these findings could have valuable implications beyond plant biology. Third, the discovery of a cell death suppression mechanism at the translational level will also challenge some common beliefs of the roles and regulation of death-promoting genes in disease development. The suppression of cell death may appear to benefit pathogen growth initially, but over time, it could reduce the selection pressure that drives the host–pathogen arms race in evolution.

In summary, Zhou and colleagues’ discovery of a phase separation control mechanism of gene translation provides a major conceptual advance in understanding the complex plant immune system. Similar to a circuit rheostat, plants appear to fine-tune the intensity of immune signaling through HEM1 phase separation to balance cell survival and death. Future research using synthetic approaches to harness HEM1’s phase behavior could have practical applications in both agriculture and human health.

## Data Availability

Not applicable.
